# Vertical integration of clinical knowledge in preclinical medical education: assessing the impact on the first-year biology curriculum in a German medical school

**DOI:** 10.1186/s12909-026-10716-7

**Published:** 2026-11-03

**Authors:** Robert-Patrick Steiner, Jean-Paul Bereuter, Maximilian Vogt, Florian Bruns, Andrea Rosner, Elisabeth Dinter, Doreen William, Jeannine Schübel, Martin Bortz, Susanne Kämmerer, Marjo Wijnen-Meijer, Lydia Günther

**Affiliations:** 1 Division of Medical Biology, Department of Psychiatry and Psychotherapy, University Hospital Carl Gustav Carus at TUD Dresden University of Technology and Faculty of Medicine of TUD Dresden University of Technology, Dresden, Germany; 2 Department of Visceral, Thoracic and Vascular Surgery, University Hospital Carl Gustav Carus at TUD Dresden University of Technology and Faculty of Medicine of TUD Dresden University of Technology, Dresden, Germany; 3 Institute for History of Medicine, Faculty of Medicine of TUD Dresden University of Technology, Dresden, Germany; 4 Department of Medicine I, University Hospital Carl Gustav Carus at TUD Dresden University of Technology and Faculty of Medicine of TUD Dresden University of Technology, Dresden, Germany; 5 Department of Neurology, University Hospital Carl Gustav Carus at TUD Dresden University of Technology and Faculty of Medicine of TUD Dresden University of Technology, Dresden, Germany; 6 Institute for Clinical Genetics, University Hospital Carl Gustav Carus at TUD Dresden University of Technology and Faculty of Medicine of TUD Dresden University of Technology, Dresden, Germany; 7 Department for Primary Care - Family Medicine, Medical University Lusatia – Carl Thiem, Cottbus, Germany; 8 Department of Family Medicine, University Hospital Carl Gustav Carus at TUD Dresden University of Technology and Faculty of Medicine of TUD Dresden University of Technology, Dresden, Germany; 9 Institute for Pharmacology and Toxicology, Faculty of Medicine of TUD Dresden University of Technology, Dresden, Germany; 10 Institute of Medical Education, Faculty of Medicine of TUD Dresden University of Technology, Dresden, Germany

**Keywords:** Medical education, Interdisciplinary education, Clinical impulses, Vertical integration

## Abstract

**Background:**

In German medical curricula, the preclinical phase is often predominantly theoretical and only weakly connected to clinical practice. Patient contact is rare, which may limit the early development of illness scripts. Consequently, students may not fully appreciate the relationships between foundational biological knowledge, disease pathophysiology, diagnosis and treatment. In this study, we implemented clinical impulse lectures within first-semester biology classes and evaluated their association with knowledge, learning strategies and perceived preparedness for clinical practice.

**Material and methods:**

First-semester medical students received the regular biology curriculum enriched with up to six clinical impulse lectures, including one session with patient contact. Data were collected using pre-intervention (*n* = 227) and post-intervention (*n* = 105) questionnaires assessing self-perceived biological and clinical knowledge. We also assessed learning motivation and strategy as well as students'' perceptions of the connection between theory and clinical practice. A 35-item multiple-choice question (MCQ) test was administered to objectively assess knowledge (*n* = 71). Each clinical impulse was additionally evaluated using a short questionnaire.

**Results:**

Students reported greater perceived preparedness regarding their biological knowledge after completing the biology course. Mean self-assessed biology knowledge improved from 2.9 to 2.3 (*p* < 0.001; 1 = best grade), and self-assessed clinical impulse-related knowledge improved from 3.3 to 2.5 (*p* < 0.001; 1 = best grade). The clinical impulses were perceived as highlighting the relevance of scientific foundations to clinical practice and as motivating integrated learning. Students who had attended more clinical impulses more frequently agreed with these statements. More than 80% supported the concept of vertical integration. In the objective MCQ assessment, students with greater exposure to clinical impulses achieved higher scores on biology questions; the difference in clinical impulse-related MCQ scores was not statistically significant.

**Conclusion:**

Early clinical impulses were well accepted by first-semester medical students. Greater exposure was associated with stronger motivation and higher biology MCQ scores, whereas the difference in clinical impulse-related MCQ performance was not statistically significant. Given the unequal exposure groups and concurrent standard biology curriculum, these findings should be interpreted as preliminary evidence of an association rather than proof of a causal effect.

**Supplementary Information:**

The online version contains supplementary material available at https://doi.org/10.1186/s12909-026-10716-7.

## Introduction

Medical curricula are often organised traditionally, with several years of basic science followed by several years of clinical science and practice. There is an ongoing discussion about how to organise medical education for the twenty-first century [1, 2]. Competency-based curricula, explicit learning objectives and physician competency frameworks have become central reference points for curricular reform [3]. Recent syntheses also show that competency-based medical education is implemented and evaluated heterogeneously across institutions [4]. In Germany, modernization efforts resulted in the "Masterplan Medizinstudium 2020" [5]. A key criticism is that preclinical education is overly theoretical and that foundational knowledge is not always adequately retained during later training [6]. Knowledge decay is a recurring concern across health-professions education. In medical students, basic-science performance can decline substantially within months after initial instruction [7]. Comparable concerns have been documented in other health professions: longitudinal anatomy knowledge loss has been reported in nursing students [8], and long-term anatomy retention has been examined in physical therapy curricula [9]. These findings provide an additional rationale for repeatedly reactivating foundational knowledge across the curriculum and linking it to clinically meaningful contexts. Moreover, the preclinical and clinical phases are largely separated by the first part of the state examination. This disconnect highlights the need for an integrated learning approach from an early stage, commonly described as vertical integration (VI). Vertically integrated curricula are characterised by early clinical experience, integration of biomedical sciences with clinical cases, longitudinal increases in clinical responsibility and an extended clerkship in the final academic year [10, 11]. Both professionals and students place high importance on competencies that characterise VI curricula, such as clinical knowledge, patient care, communication and collaboration. VI curricula may support professional development [12], and problem-based learning, as well as the clinical relevance of course content, may improve preparedness for medical practice [13]. Recent meta-analyses of problem-based learning in medical and nursing education report benefits for learner satisfaction and aspects of clinical competence, whereas effects on theoretical or factual knowledge are less consistent [14, 15]. Efforts to expose medical students to clinical situations as early as possible differ between countries [16]. Implementation requires comprehensive structural changes and a well-planned change process [17], including staff training and a sustained commitment to re-evaluation and revision [18]. Dahle et al. (2002) highlighted the challenges posed by such structural changes [19], and several barriers (e.g. appropriate assessment) and facilitators (e.g. continuity of supervision and a safe, supportive learning environment) have been reported during implementation of longitudinal integrated curricula [20]. Despite the growing global popularity of vertical integration, broad implementation remains limited at German medical faculties. German model curricula have nevertheless reported successful implementation of such concepts [21, 22]. In 2003, RWTH Aachen also developed a restructured medical curriculum and implemented principles of VI [23]. Implementation took several years and faced numerous challenges, particularly the high resource demands of small-group learning and the need for continuous curriculum management and refinement. To facilitate the introduction of VI at medical faculties, gradual incorporation of clinical elements into the early curriculum, such as early clinical exposure (ECE), may provide a feasible first step [24]. This approach introduces clinical elements and practice early in the preclinical phase to bridge the gap between basic sciences and clinical care, potentially enhancing students'' understanding and retention of foundational knowledge while fostering clinical reasoning from the beginning of medical training [25–27].

The implementation of the National Competence-Based Catalogue of Learning Objectives for Undergraduate Medical Education (NKLM) [28] at the Medical Faculty Dresden (MFD) provides momentum to redesign the traditional preclinical curriculum using an interdisciplinary, systems-based approach that links the basic sciences more closely with clinical subjects. Such curricular changes should be accompanied by systematic evaluation. As Brauer et al. (2015) note, evaluating vertical integration is important, although establishing a truly controlled curriculum trial is difficult [10]. Hirsh et al. (2012) [29] showed that students trained in a longitudinal integrated clerkship curriculum performed better than traditionally trained students in clinical knowledge and skills. Students following an integrated curriculum achieved significantly higher scores in basic and clinical knowledge domains on the Dutch inter-university progress test than students following a conventional curriculum [30].

Our objectives were to assess whether integrated teaching and early clinical exposure were associated with students'' self-perceived biological knowledge, motivation, perceived preparedness for clinical studies and performance on standardised multiple-choice questions (MCQs). Using biology within a traditionally organised curriculum as an example, we sought to provide empirical evidence on the feasibility and potential educational value of early clinical exposure in preclinical education and to contribute to the broader discussion of curriculum reform in German medical education and beyond.

## Material and methods

A prospective interventional study with pre- and post-intervention evaluation was conducted to assess the impact of clinical impulse lectures on first-semester medical students'' biological knowledge, motivation and perceived preparedness for further clinical studies.

### Study population

All first-semester medical students enrolled in the regular biology curriculum were eligible to participate in the study. The clinical impulses were embedded into regularly scheduled biology teaching sessions within the compulsory curriculum. Students did not actively select individual clinical impulse topics. Due to timetabling constraints and the availability of the clinicians delivering the sessions, not every student group received every clinical impulse. Consequently, individual exposure ranged from 0 to 6 clinical impulses. In the final questionnaire, students reported which specific clinical impulses they had attended. These data allowed the number and combination of clinical impulses received by each respondent to be determined and were used for subsequent subgroup analyses.

The study cohort comprised all first-semester students (*N* = 227) who completed the baseline questionnaire at the beginning of the semester during practical training. In accordance with the study design, participants were exposed to up to six different clinical impulse lectures. After completion of the curriculum, 105 students (46% of the initial cohort) completed the final questionnaire. Of these, 71 (31.3% of the initial cohort) also completed the MCQ-based assessment covering biological and clinical impulse-related topics. Participants in the baseline, final and MCQ samples were similar in age (baseline: 22.1 years; final: 22.3 years; MCQ: 22.5 years), sex (male: 32.6%, 28.6% and 32.4%, respectively) and high-school grade point average (HS-GPA; baseline: 1.61; final: 1.58; MCQ: 1.60) (Table 1).

**Table 1:** Participant characteristics. Comparison of participant characteristics across the baseline, final and multiple-choice question (MCQ) samples

Item	Baseline (*n* = 227)	Final (*n* = 105)	MCQs (*n* = 71)
Age, mean (SD), years	22.1 (3.7)	22.3 (3.3)	22.5 (3.5)
Male sex, n (%)	74 (32.6)	30 (28.6)	23 (32.4)
HS-GPA, mean (SD)	1.61 (0.55)	1.58 (0.56)	1.6 (0.55)
Previous medical education, n (%)	130 (57.3)	54 (51.4)	39 (54.9)
Biology at school, n (%)			
Basic level	72 (31.7)	47 (44.7)	31 (43.7)
Advanced level	113 (49.8)	48 (45.7)	32 (45.1)
Neither basic nor advanced	17 (7.5)	3 (2.9)	3 (4.2)
Not during A-levels	12 (5.3)	7 (6.7)	5 (7)
Other	13 (5.7)	0 (0)	0 (0)

### Context

The regular first-semester biology curriculum comprised teaching on living systems and the characteristics of life, protein structure and function, DNA, chromosomes and inheritance, blood cells and the glycocalyx, bacteria and antibiotics, and parasitology. The corresponding practical sessions formed part of the compulsory curriculum for all students and included basic topic-specific biological exercises, such as microscopy and simple experiments. The six clinical impulses were thematically linked to these curriculum topics and were scheduled directly after the corresponding regular biology lecture and practical session, ensuring that the relevant theoretical foundations had already been covered. The clinical topics were selected jointly by the biology course supervisor and the study team using a pragmatic curriculum-development approach. Selection was guided by two main criteria: a clear and clinically relevant connection to the corresponding established biology topic and the feasibility of involving clinicians with appropriate expertise who were available to contribute to the course. Thus, the selected impulses were intended as clinically meaningful examples that could be integrated into the existing curriculum rather than as an exhaustive or formally prioritised set of clinical topics. All clinical impulses were designed as interactive lectures rather than uninterrupted didactic presentations. Clinicians were instructed to facilitate active student participation through guided discussion and joint analysis of real or modelled clinical situations, prompting students to apply the biological concepts taught immediately beforehand. In the neurodegenerative disease impulse, this approach was supplemented by a live interview with a patient with Parkinson''s disease. Observable clinical manifestations were directly related to the underlying biological mechanisms, enabling students to connect foundational biological knowledge with the patient''s physical findings. Each clinical impulse lasted 30–45 min and was integrated into the regular teaching schedule. No additional preparatory material was provided specifically for the clinical impulses, and students were not restricted from undertaking individual self-study. The clinical impulses focused on applying previously taught biological concepts to clinical contexts (Table 2; detailed learning objectives are provided in Supplementary Table 1).

**Table 2:** Overview of biology lectures and corresponding clinical impulses. According to the biology curriculum, six clinical impulse lectures were designed and integrated into the curriculum. The table shows the clinical field and didactic method used for each clinical impulse

Biology	Clinical impulse	Clinical field	Didactic method
Living systems and characteristics of life	Ethics and medicine	History, Theory and Ethics of Medicine	Interactive lecture
Proteins: structure and function	Neurodegenerative diseases	Neurology	Interactive lecture and patient interview
DNA, chromosomes and inheritance	Cytogenetics	Human genetics	Interactive lecture and video of genetic methods
Blood cells and glycocalyx	Transfusion medicine	Transfusion medicine	Interactive lecture
Bacteria and antibiotics	Antibiotics	Pharmacology	Interactive lecture
Parasitology	Clinical parasitology	Family medicine	Interactive lecture

### Data collection and intervention

**Fig. 1:**
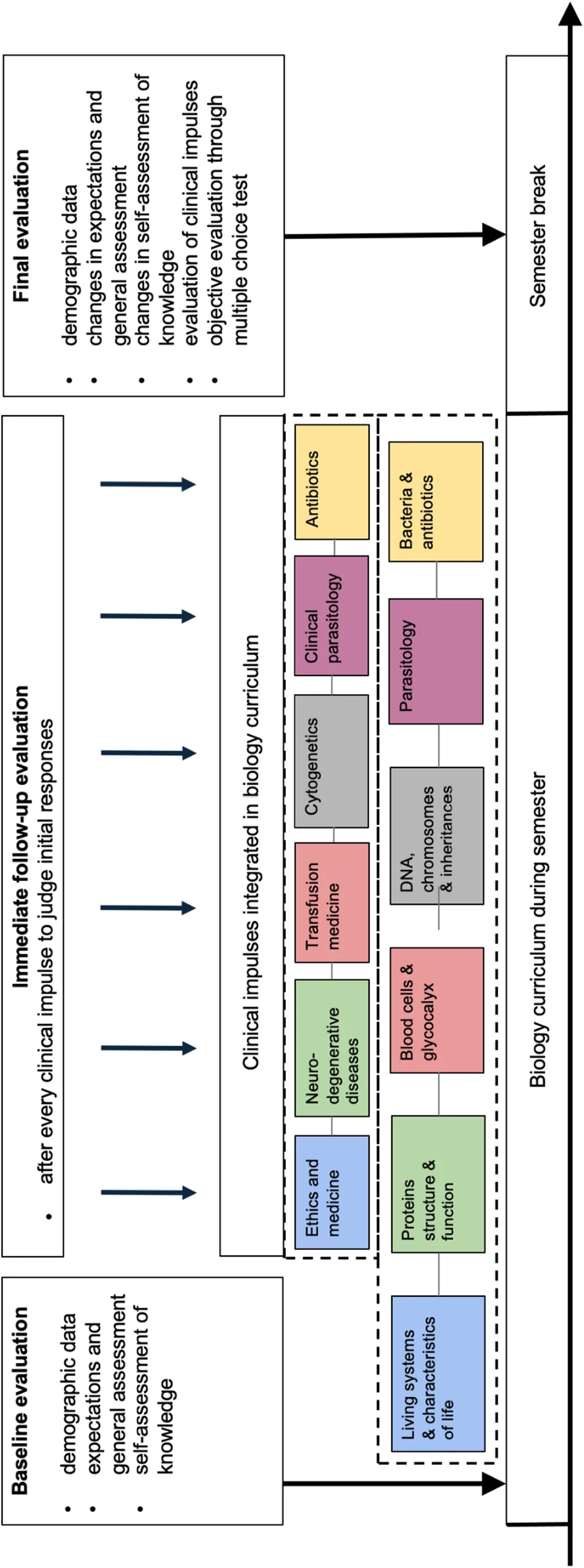
Timeline of the project. Schematic overview of the curriculum showing the biology lectures, integrated clinical impulse lectures and evaluation time points

Data were collected at different time points using standardised questionnaires (Fig. 1). Participants completed questionnaires before the curriculum (baseline, *n* = 227) and after the curriculum (final, *n* = 105). All respondents to the final questionnaire (*n* = 105) reported the number of clinical impulse lectures they had attended. Of these, 94 had attended four to six impulses and 11 had attended three or fewer. Of the 105 respondents, 71 also completed the MCQ test (64 in the four-to-six-impulse group and 7 in the zero-to-three-impulse group). The final questionnaire therefore included students with different levels of exposure to the clinical impulses. To examine associations between exposure and subjective and objective outcomes, subgroup analyses were conducted according to these two exposure groups (group 1: 0–3 impulses, *n* = 11 of 105; group 2: 4–6 impulses, *n* = 94 of 105). In addition, short follow-up questionnaires were administered directly after each clinical impulse to assess immediate student feedback. The questionnaires were developed specifically for this study by the authors in an iterative, collaborative process. All items were reviewed by scientists and collaborating medical students to estimate completion time and remove ambiguity. The questionnaires were distributed using LimeSurvey 3 and are provided in the Supplementary Materials. The baseline assessment comprised three domains. We collected demographic information, previous biological education, self-assessed biological skills (both overall and in specific fields covered by the clinical impulses), and expectations regarding the biology curriculum. The baseline questionnaire consisted of 12 items. In addition to the baseline items, the follow-up questionnaire contained 10 additional items assessing attendance and the overall reception of the clinical impulses. A multiple-choice competency test comprising 35 MCQs (17 basic biology and 18 clinical knowledge questions) was included to objectively assess knowledge after participation in the biology course and clinical impulses (Cronbach''s alpha: 0.7, 95% CI = 0.57–0.80). All items were single-best-answer questions and the test covered preclinical and clinical knowledge across all six topics addressed in the mandatory lectures and practical training and in the clinical impulses. Each MCQ followed the structure and difficulty level of the first part of the German State Examination, which all students take after the fourth semester. The MCQ were developed by the lecturers and clinicians responsible for each topic and reviewed by the study team within an iterative process. An overview of the number of biology and clinical impulse related questions can be found in Table 3. Completing the questionnaire took approximately 30 min. Although attendance at individual clinical impulses and item-level MCQ responses were available, the resulting topic-specific attendance groups were small and unevenly distributed and were therefore considered insufficient for robust inferential comparisons. Subgroup analyses were consequently based on overall exposure to the clinical impulses (0–3 versus 4–6 impulses).

**Table 3:** Distribution of the analysed MCQ items across curriculum topics and subscales

Curriculum topic/clinical impulse	Biology items	Clinical impulse items	Total
Living systems/Ethics and medicine	2	3	5
Proteins/Neurodegenerative diseases	2	3	5
Blood cells and glycocalyx/Transfusion medicine	6	3	9
DNA, chromosomes and inheritance/Cytogenetics	2	3	5
Parasitology/Clinical parasitology	2	3	5
Bacteria & antibiotics/Antibiotics	3	3	6
**Total**	**17**	**18**	**35**

### Data analysis and statistics

Statistical analyses were conducted using GraphPad Prism version 10. Normality of continuous data was assessed using the Kolmogorov–Smirnov test and inspection of frequency distributions. Continuous data are presented as means with standard deviations (SD), and categorical data as frequencies. Depending on the data characteristics, appropriate statistical tests were used (unpaired Student''s *t*-test and analysis of variance [ANOVA]) for between-group comparisons. p values < 0.05 were considered statistically significant.

### Ethics approval and consent to participate

Participation in the evaluations was voluntary but incentivised through voucher raffles. The study adhered to the Declaration of Helsinki. In accordance with Saxon law, this anonymous cross-sectional survey did not fall within the scope of mandatory review by an ethics committee. The Ethics Committee at the TUD Dresden University of Technology (TUD) is established under § 9(4) of the Saxon Healthcare Professions Chamber Act (Sächsisches Heilberufekammergesetz, SächsHKaG). Under § 15(1) of the Professional Code of the Saxon State Chamber of Physicians (Berufsordnung der Sächsischen Landesärztekammer), which is issued on the basis of the SächsHKaG, physicians must seek advice from an ethics committee only for research that intervenes in a person''s physical or psychological integrity, or that uses bodily material or data that can be traced to an identifiable individual. Our study met neither condition. Data were collected anonymously, no personally identifiable information was processed, and participation involved no physical or psychological intervention. The TUD Ethics Committee''s own guidance also states that surveys based solely on anonymous data collection do not meet the minimum requirements for a research project it must review. All participants gave informed consent electronically before enrolment and agreed to the applicable data-protection provisions. Questionnaire responses were stored on university servers. The study followed the data privacy rules of the Medical Faculty Dresden (MFD) and TUD. Clinical trial number: not applicable.

## Results

### Changes in student expectations and preparedness

**Fig. 2:**
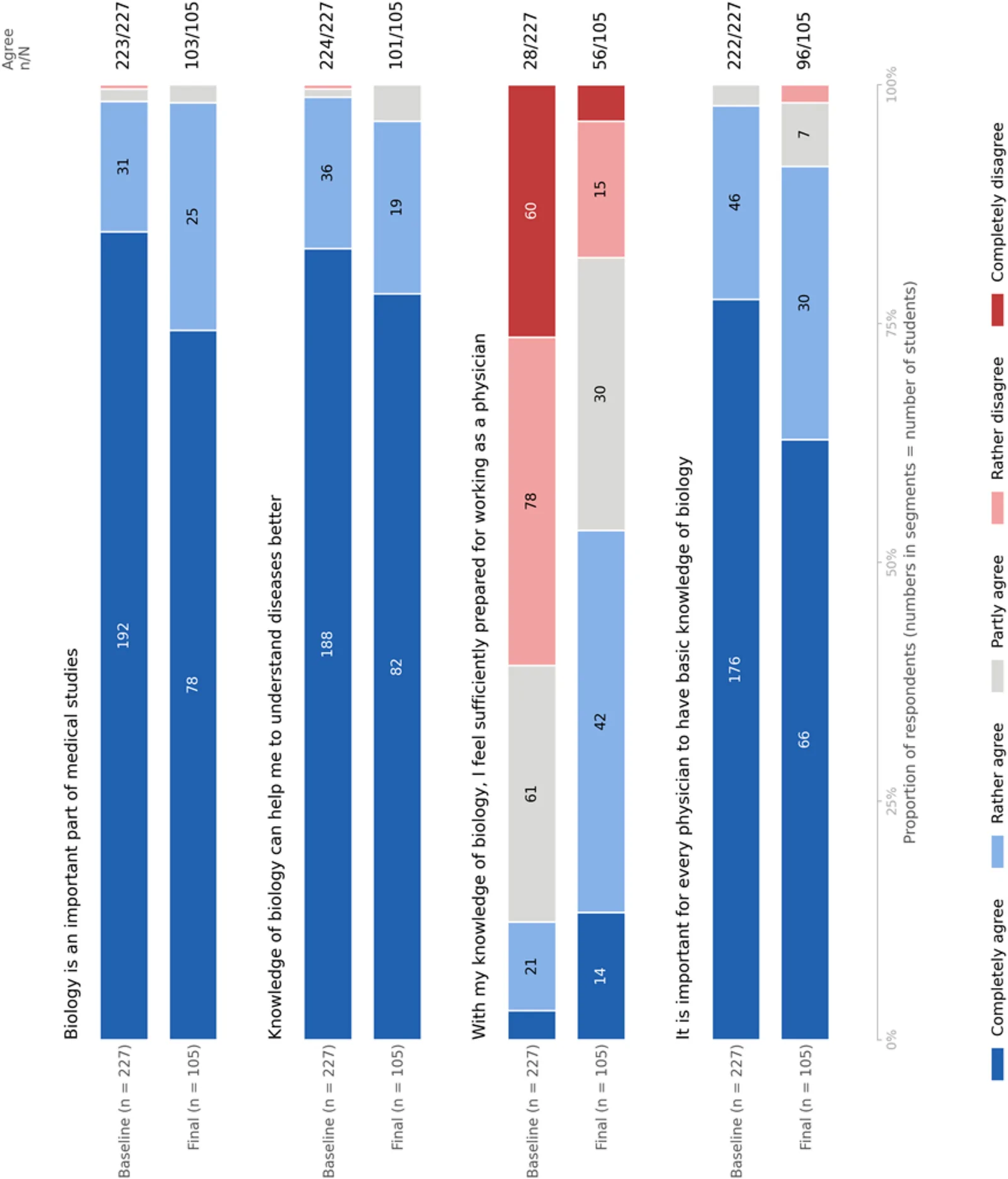
Students'' initial and final perceptions of the relevance of biology in medical studies. Comparison of students'' responses before (baseline) and after (final) the curriculum containing clinical impulses. Numbers within bar segments indicate the number of students; the right column shows the number of students who agreed (''completely agree'' or ''rather agree'') relative to all respondents (n/N)

At the beginning of the semester, most students reported that their biological knowledge left them insufficiently prepared for their future work as physicians. The proportion reporting sufficient preparedness was higher after completion of the biology curriculum with integrated clinical impulses. While only 12% of students felt sufficiently prepared at baseline, 53% reported feeling well prepared in the final survey (Fig. 2). This suggests greater perceived readiness and confidence after the semester. The perceived importance of biology overall did not differ substantially between the baseline and final assessments. After the curriculum, students showed a slight tendency to rate biological knowledge as less important for becoming a physician.

### Subjective assessment of knowledge

**Fig. 3:**
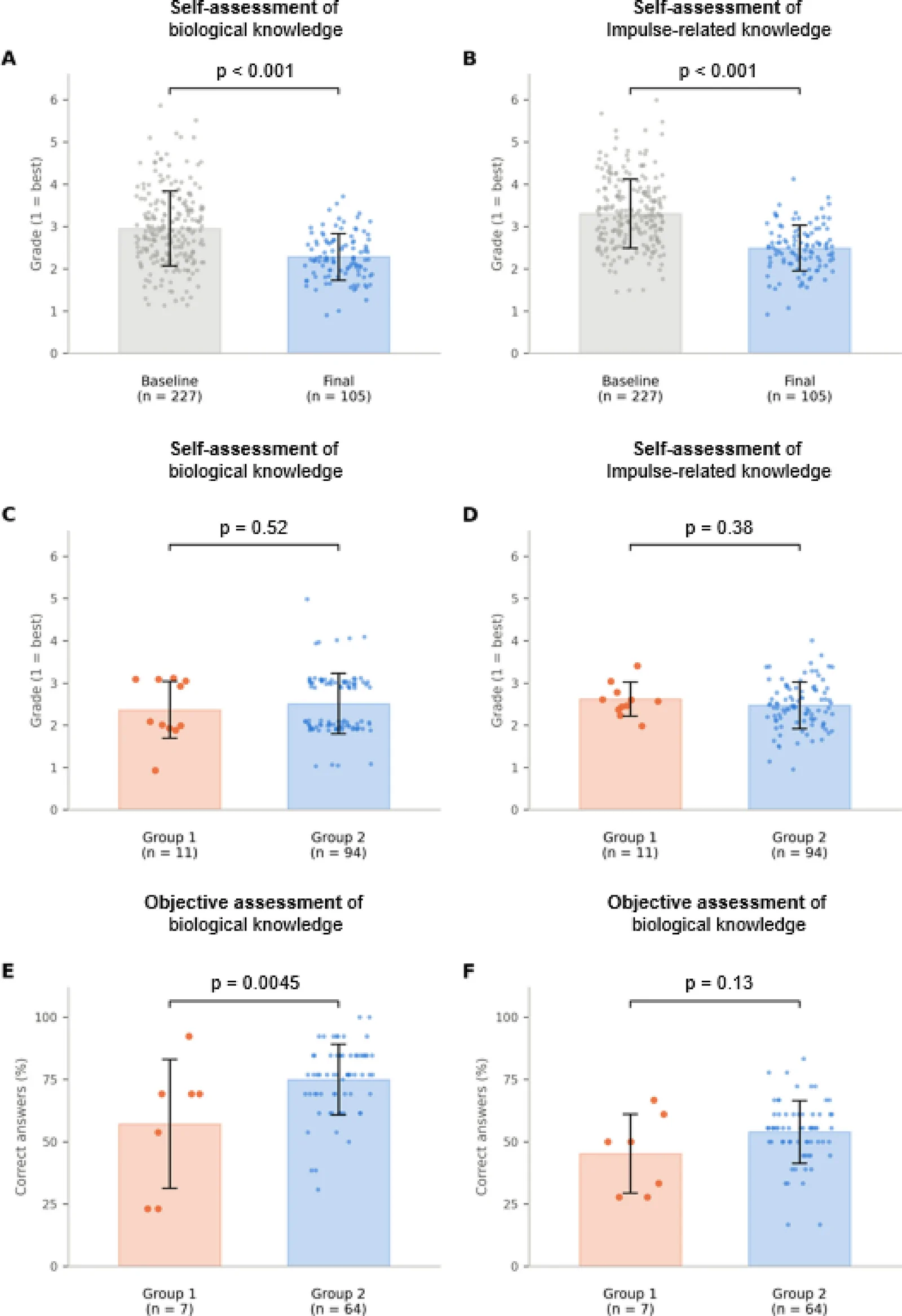
Impact of the curriculum on the students’ self-assessed and objective knowledge. The figure shows mean values with standard deviations (**A**–**D**) and percentages of correct responses with dispersion measures (**E**–**F**). **A** Self-assessed biological knowledge at baseline and final evaluation. **B** Self-assessed clinical impulse-related knowledge at baseline and final evaluation. **C** Comparison of self-assessed biology knowledge between group 1 (0–3 clinical impulses attended) and group 2 (4–6 clinical impulses attended) at final evaluation. **D** Comparison of self-assessed clinical impulse-related knowledge between group 1 (0–3 clinical impulses attended) and group 2 (4–6 clinical impulses attended) at final evaluation. **E** Comparison of biology MCQ scores between group 1 (0–3 clinical impulses attended) and group 2 (4–6 clinical impulses attended). **F** Comparison of clinical impulse-related MCQ scores between group 1 (0–3 clinical impulses attended) and group 2 (4–6 clinical impulses attended). Statistical analysis was conducted using an unpaired Student''s t-test

Students assessed their knowledge using the German grading scale (1 = best, 6 = worst). At baseline, students rated their clinical knowledge lower than their biology knowledge (biology: 2.9 vs. clinical: 3.3, *p* < 0.001), as shown in Supplementary Fig. 1. Self-assessed biology knowledge improved over the semester (Fig. 3A, Supplementary Fig. 2). Students rated their understanding of biological topics significantly better after completing the biology curriculum and clinical impulse lectures than at baseline (2.9 vs. 2.3, *p* < 0.001). Similarly, self-assessed knowledge of the topics covered in the clinical impulses improved from 3.3 to 2.5 (*p* < 0.001), as shown in Fig. 3B and Supplementary Fig. 3.

To explore whether the number of clinical impulses attended was associated with self-assessed knowledge, subgroup analyses were conducted. The cohort was divided into two groups according to overall exposure: group 1 attended 0—3 clinical impulses (*n* = 11), and group 2 attended 4—6 clinical impulses (*n* = 94). The groups were similar in baseline participant characteristics (Supplementary Table 2). No significant differences were observed between the groups in self-assessed biology or clinical impulse-related knowledge (biology: group 1, 2.46 vs. group 2, 2.51, *p* = 0.52; clinical impulse-related knowledge: group 1, 2.62 vs. group 2, 2.47, *p* = 0.38; Fig. 3C, D).

### Objective knowledge assessment

Out of the 105 respondents of the final questionnaire, 71 completed the voluntary MCQ section, resulting in *n* = 7 responses for group 1 and *n* = 64 responses for group 2. MCQ completion was thus similar in both exposure groups (group 1: 7 of 11, 64%; group 2: 64 of 94, 68%). When analyzing the objective knowledge, assessed by MCQ performance, the exposure groups differed significantly for biology questions (Fig. 3E, F). Students who attended 4–6 clinical impulses (group 2, *n* = 64) achieved higher scores on biology MCQs than those who attended only 0–3 impulses (group 1, *n* = 7) (group 1: 57.1%; group 2: 75.3%; *p* = 0.0045). Clinical impulse-related MCQ scores were also numerically higher in the greater-exposure group, but the difference was not statistically significant (group 1: 45.2%; group 2: 53.3%; *p* = 0.13). Overall, it appears that basic sciences taught in a clinical context led to better knowledge outcomes. However, given the small size of group 1, these differences should be interpreted with caution.

### Student evaluation of the curriculum

**Fig. 4:**
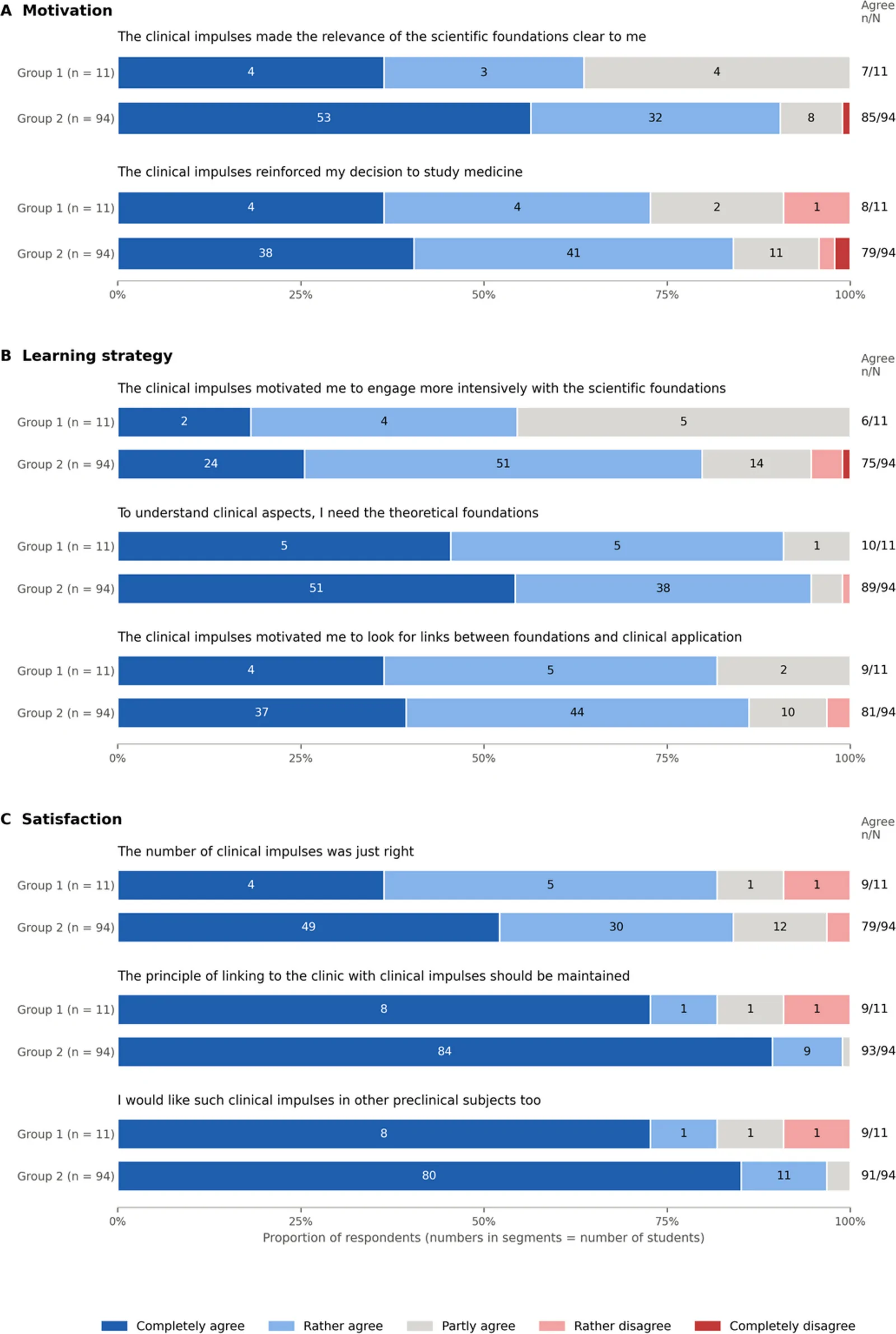
Evaluation of the curriculum by participants. Comparison of responses between group 1 (0–3 clinical impulses attended) and group 2 (4–6 clinical impulses attended). Stacked bars show the proportion of respondents; numbers within bar segments indicate the number of students. The right column shows the number of students who agreed (''completely agree'' or ''rather agree'') relative to all respondents in each group (n/N)

The evaluation addressed three domains. First, student motivation was assessed (Fig. 4A). Students who attended 4–6 clinical impulses more frequently agreed that the impulses motivated them to learn the scientific foundations needed to understand disease pathophysiology than students who attended 0–3 impulses (group 1: 7/11, 64%; group 2: 85/94, 90%). Second, learning strategy was assessed (Fig. 4B). Students with greater exposure more frequently reported engaging more deeply with the scientific foundations of disease (group 1: 6/11, 55%; group 2: 75/94, 80%). Third, satisfaction with the curriculum was assessed (Fig. 4C). Students in both groups were highly satisfied with the number of clinical impulses (group 1: 9/11, 82%; group 2: 79/94, 84%) and supported maintaining the integrated clinical impulse concept (group 1: 9/11, 82%; group 2: 93/94, 99%). Most students also wanted clinical impulses to be incorporated into other preclinical subjects (group 1: 9/11, 82%; group 2: 91/94, 97%).

### Student evaluation of clinical impulse lectures

**Fig. 5:**
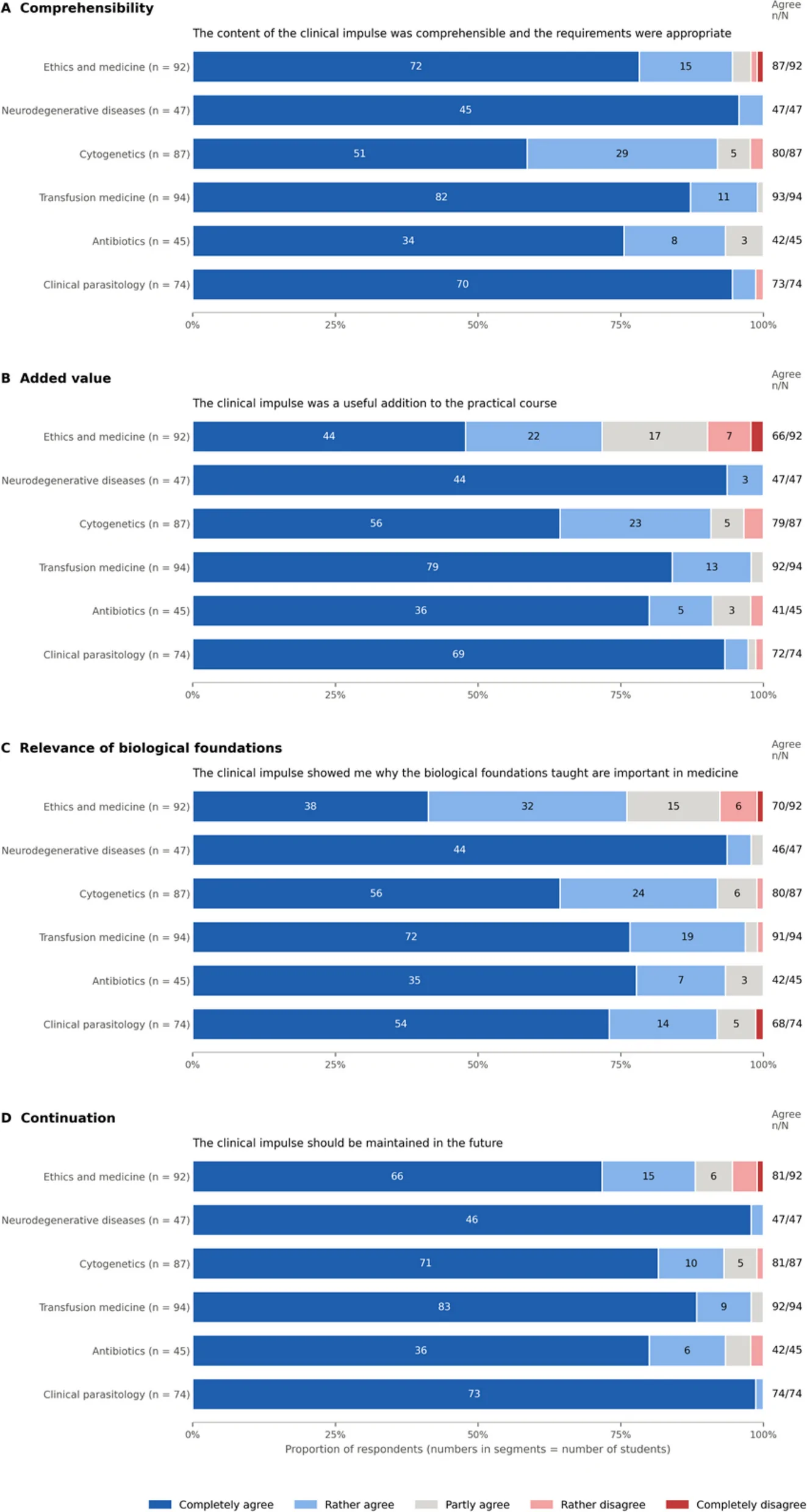
Evaluation of the clinical impulse lectures. Each clinical impulse was evaluated using four questions; n indicates the number of students who evaluated the respective impulse. Stacked bars show the proportion of respondents; numbers within bar segments indicate the number of students, and the right column shows the number of students who agreed (''completely agree'' or ''rather agree'') relative to all respondents (n/N)

Students evaluated the clinical impulse lectures at the end of the semester. As shown in Fig. 5, a substantial majority of respondents agreed (''completely agree'' or ''rather agree'') with statements regarding the comprehensibility, value and educational impact of the clinical impulses. More than 80% found the content comprehensible and the level of difficulty appropriate across several topics, including neurodegenerative diseases, clinical parasitology and cytogenetics. A similar proportion perceived the clinical impulses as a valuable addition to the biology course, with the highest ratings for the neurodegenerative disease and clinical parasitology impulses. Students also generally agreed that the clinical impulses helped them understand the importance of biological foundations in medicine, with particularly strong agreement for neurodegenerative diseases, cytogenetics and antibiotics. Overall, respondents strongly supported maintaining the clinical impulses in future curricula.

## Discussion

The present study evaluated six clinical impulse lectures integrated into the first-semester biology curriculum as a pragmatic form of early clinical exposure. Students reported greater perceived preparedness and better self-assessed biological and clinical impulse-related knowledge after the semester. Greater exposure was also associated with stronger motivation to engage with the scientific foundations of disease. In the objective assessment, students who attended four to six impulses achieved higher biology MCQ scores than those who attended zero to three impulses, whereas the difference in clinical impulse-related MCQ scores was not statistically significant. These findings suggest that the approach was feasible, well accepted, and potentially educationally beneficial, but they do not establish causality because the impulses were embedded within the compulsory biology curriculum.

Self-assessed biology knowledge improved by 0.6 grade points, and clinical impulse-related knowledge by 0.8 grade points. Similar positive changes in attitudes and perceived preparedness have been reported for early clinical exposure [31]. However, self-assessed knowledge did not differ significantly between the two exposure groups, and self-assessment during the first months of medical school may be imprecise [32]. These outcomes should therefore be interpreted primarily as changes in perceived preparedness rather than direct evidence of knowledge gain. The perceived importance of biology itself also did not increase over the semester [33].

The MCQ results provide a complementary objective measure. Biology scores were 75.3% in the higher-exposure group and 57.1% in the lower-exposure group (*p* = 0.0045), while clinical impulse-related scores were 53.3% and 45.2%, respectively (*p* = 0.13). These findings are compatible with previous reports linking early clinical exposure or integrated curricula with basic-science learning performance [30, 34]. However, the MCQ groups were highly unequal (*n* = 64 versus *n* = 7), and only 71 of 227 baseline participants completed the MCQ assessment. Although individual impulse attendance could be linked to item-level responses, the topic-specific attendance groups were too small and uneven for reliable inferential analysis. We therefore focused on overall exposure and cannot determine whether individual impulse topics differed in their educational effects.

The clinical impulses were positively evaluated, with more than 80% of respondents supporting continuation of the concept. Students with greater exposure more frequently reported deeper engagement with the scientific foundations of disease, consistent with previous reports of positive attitudes towards early clinical exposure and patient contact [35–39]. The live patient session involving a person with Parkinson''s disease illustrates the intended mechanism: observable physical manifestations were directly linked to the biological concepts taught immediately beforehand. Patient contact may therefore make the relevance of foundational science more tangible, although its specific contribution could not be isolated in this study.

The main limitations are the single-centre setting, the integration of the impulses within the standard curriculum, scheduling-based rather than experimentally balanced exposure, unequal subgroup sizes, and substantial attrition from baseline to final and MCQ assessments. These factors limit causal interpretation and statistical power. Future studies should use prospectively balanced comparison groups, larger objective assessments and longer follow-up to examine knowledge retention, later clinical competence and the relative value of different impulse topics.

## Conclusion

Brief clinical impulses delivered directly after corresponding biology teaching were feasible and well accepted by first-semester medical students. Greater exposure was associated with stronger motivation and higher biology MCQ scores, while the difference in clinical impulse-related MCQ performance was not statistically significant. Given the unequal exposure groups, attrition and concurrent standard biology curriculum, the results should be interpreted as preliminary evidence rather than proof of a causal effect. Controlled studies with balanced groups and longer-term follow-up are needed.

## Supplementary Information

Supplementary Material 1.

Supplementary Material 2.

Supplementary Material 3.

Supplementary Material 4.

## Data Availability

Data is provided within the manuscript or supplementary information files respective made available on request by the authors.

## Abbreviations

ECE: Early clinical exposure
HS-GPA: High-school grade point average
MCQ: Multiple-choice question
MFD: Medical Faculty Dresden
PBL: Problem-based learning
TUD: TUD Dresden University of Technology
VI: Vertical integration
