# Supplementary material for: Vertical integration of clinical knowledge in preclinical medical education: assessing the impact on the first-year biology curriculum in a German medical school
Source: BMC Med Educ. 2026 Nov 3;26:1774. doi: 10.1186/s12909-026-10716-7 (PMC13729362; doi:10.1186/s12909-026-10716-7)
Supplement: Supplementary file 1 [file 12909_2026_10716_MOESM1_ESM.pdf]

Basic participant characterization of the study cohort

1. **How old are you?**

2. **Which sex do you assign yourself to?**
   a) Male
   b) Female
   c) Other

3. **What was your high school GPA?**

4. **Did you receive training or a different degree in a medical field before enrolling into medical school?**
   Yes / No

   If yes:
   Which training/degree did you complete?
   [open text field]

Biological Background

5. **On what level did you participate in biology at school?**
   a) Basic level
   b) Advanced level
   c) Biology was not separated into basic nor advanced courses
   d) I dropped Biology during my A-levels
   e) Other [open text field]

6. **How would you self-assess your knowledge of biology after having completed the courses?**
   (Please use German school grades 1–6, where 1 = best, 6 = worst)

7. **How would you self-assess your knowledge in the following biological-medical subject areas?**
   (Please use German school grades 1–6, where 1 = best, 6 = worst)

   - Living systems / Characteristics of life
   - Ethics of life / Ethics and medicine
   - Proteins and protein folding
   - Neurodegenerative diseases / Parkinson’s disease
   - Glycocalyx and ABO blood group system
   - Blood transfusions and bedside test
   - Fundamentals of human genetics / The human genome
   - Cytogenetic diagnostics / Methods in human genetics
   - Bacterial structure and bacterial pathogenicity
   - Antibiotics
   - General parasitology
   - Medically relevant diseases – Scabies
   - Medically relevant diseases – Malaria
   - Medically relevant diseases – Lyme disease

Expectations

Please rate the following statements using:
[completely agree / rather agree / partly/partly / rather disagree / completely disagree]

1. Biology is an essential part of medical studies.
2. Biology helps to better understand diseases.
3. I feel sufficiently prepared to work as a doctor with my knowledge in biology.
4. It is important for every doctor to have a basic knowledge of biology.
