# Supplementary material for: Vertical integration of clinical knowledge in preclinical medical education: assessing the impact on the first-year biology curriculum in a German medical school
Source: BMC Med Educ. 2026 Nov 3;26:1774. doi: 10.1186/s12909-026-10716-7 (PMC13729362; doi:10.1186/s12909-026-10716-7)
Supplement: Supplementary file 2 [file 12909_2026_10716_MOESM2_ESM.pdf]

Basic participant characterization of the study cohort

1. **How old are you?**

2. **Which sex do you assign yourself to?**
   a) Male
   b) Female
   c) Other

3. **What was your high school GPA?**

4. **Did you receive training or a different degree in a medical field before enrolling into medical school?**
   Yes / No

   If yes:
   Which training/degree did you complete?
   [open text field]

Biological Background

5. **On what level did you participate in biology at school?**
   a) Basic level
   b) Advanced level
   c) Biology was not separated into basic nor advanced courses
   d) I dropped Biology during my A-levels
   e) Other [open text field]

6. **How would you self-assess your knowledge of biology after having completed the courses?**
   (Please use German school grades 1–6, where 1 = best, 6 = worst)

7. **How would you self-assess your knowledge in the following biological-medical subject areas?**
   (Please use German school grades 1–6, where 1 = best, 6 = worst)

   - Living systems / Characteristics of life
   - Ethics of life / Ethics and medicine
   - Proteins and protein folding
   - Neurodegenerative diseases / Parkinson's disease
   - Glycocalyx and ABO blood group system
   - Blood transfusions and bedside test
   - Fundamentals of human genetics / The human genome
   - Cytogenetic diagnostics / Methods in human genetics
   - Animal models in medical research
   - Bacterial structure and bacterial pathogenicity
   - Antibiotics
   - General parasitology
   - Medically relevant diseases – Scabies
   - Medically relevant diseases – Malaria
   - Medically relevant diseases – Lyme disease

Expectations and reflection

Please rate the following statements using:
[completely agree / rather agree / partly/partly / rather disagree / completely disagree]

1. Biology is an essential part of medical studies.
2. Biology helps to better understand diseases.
3. I feel sufficiently prepared to work as a doctor with my knowledge in biology.
4. It is important for every doctor to have a basic knowledge of biology.

1.4. Clinical Impulses

I attended the following clinical contributions/“impulses”:
1.4.1. Ethics and medicine – Prof. Bruns: yes / no
1.4.2. Transfusion medicine – Dr. Rosner: yes / no
1.4.3. Neurodegenerative diseases – Dr. Dinter: yes / no
1.4.4. Cytogenetics – Dr. Arlt or Dr. Grüneis: yes / no
1.4.5. Clinical parasitology – Dr. Schübel: yes / no
1.4.6. Antibiotics – Dr. Kämmerer: yes / no

1.5. Motivation and vertical integration

Please rate the following statements using:
[completely agree / rather agree / partly/party / rather disagree / completely disagree]

1.5.1. The clinical impulses have reinforced my decision to study medicine.
1.5.2. The clinical impulses made me realize the relevance of the scientific foundations in my studies.
1.5.3. For understanding clinical aspects, I need the theoretical foundations.

1.6. Learning strategy

Please rate the following statements using:
[completely agree / rather agree / partly/partly / rather disagree / completely disagree]

1.6.1. The clinical impulses have motivated me to engage more deeply with the scientific foundations.
1.6.2. The clinical impulses have motivated me to seek connections between scientific foundations and clinical application.

1.7. Evaluation and curricular impact

Please rate the following statements using:
[completely agree / rather agree / partly/partly / rather disagree / completely disagree]

1.7.1. The number of clinical impulses was just right.
1.7.2. The principle of integration with clinical experiences should be maintained.
1.7.3. I would like to have such clinical experiences in other preclinical subjects as well.
1.7.4. I could imagine the following clinical impulses as part of the biology courses: [open text field]

Objective assessment of biology knowledge - Multiple-Choice Questions

Which statement about hydrogen bonds is correct?
A) They do not occur between water molecules.
B) They are covalent and therefore strong chemical bonds.
C) They do not play a role in the formation of the DNA double helix.
D) They play a role in how fatty acid chains assemble in membranes.
E) They affect the boiling point of an aqueous solution.

Living systems are characterized by certain features. Which combination of features is found in all living systems?
A) The presence of multicellularity and an autonomous metabolism
B) The presence of cell organelles and a structural-and-functional blueprint
C) The presence of evolutionary adaptation and a structural-and-functional blueprint
D) The presence of cell organelles and an autonomous metabolism
E) The presence of evolutionary adaptation and a chemical equilibrium

A 30-year-old woman seeks information about pregnancy termination in Germany. Which of the following statements most accurately describes the legal requirements for performing an abortion within the first 12 weeks of pregnancy?
A) It is legal within the first 12 weeks if medically indicated and performed by a specialist.
B) It is legal after a state-recognized consultation and a mandatory waiting period of at least three days, without needing a specific reason.
C) It is legal if, in addition to state-recognized consultation, a psychological evaluation confirms the woman's capacity for decision-making.
D) It can be carried out without any further requirements if it occurs within the first 9 weeks after conception.
E) It is always legal once comprehensive medical and psychosocial counseling has been completed.

In the ethical debate regarding the protection of human embryos, the so-called SKIP arguments are also used. What does the SKIP acronym stand for?
A) Species, Continuity, Identity, and Potentiality
B) Species, Continuity, Integrity, and Potentiality
C) Species, Congruence, Identity, and Permanence
D) Specificity, Continuity, Integrity, and Potentiality
E) Specificity, Congruence, Identity, and Permanence

According to current medical understanding, when does human life begin?
A) With the fusion of sperm and egg cell
B) With the implantation (nidation) of the fertilized egg in the uterine wall
C) With the onset of the embryonic heartbeat in the 5th week of pregnancy
D) With the fetus's extrauterine viability at about the 22nd week of pregnancy
E) It cannot be answered through scientific means alone because individual and societal values also play a role

Proteins consist of an amino acid chain (primary structure) that is folded in three dimensions. Which statement about protein folding is correct?

A) Chaperones always fold proteins correctly with the consumption of glutathione.
B) Misfolded proteins in the cytosol are typically secreted by exocytosis.
C) Proteins are folded by proteasomes.
D) Generally, a protein begins to fold before ribosomal synthesis is complete.
E) Disulfide bonds are characteristic of the secondary structure.

An important secondary structure in proteins is the alpha-helix. Which amino acid is considered a helix breaker because it cannot be stabilized by hydrogen bonds in the alpha-helix?

A) Alanine
B) Valine
C) Methionine
D) Proline
E) Cysteine

In Parkinson's disease, misfolded proteins play a central role in its pathogenesis. Which of the following proteins is most strongly associated with Parkinson's and is a key component of Lewy bodies characteristic of this disease?

A) Tau protein
B) Beta-amyloid
C) Prion protein
D) Huntingtin
E) α-Synuclein

A 58-year-old man reports a progressive slowing of his movements over the past months. He notices stiffness in his arms and pain in the shoulder/neck area that affect his daily activities. Which additional symptom, if reported, would most strongly suggest early-stage Parkinson's disease?

A) A slight but consistent reduction in handwriting size (micrographia)
B) An occasional fine tremor of the hand when holding objects, worsening under stress
C) A sudden, unexplained loss of smell prior to the onset of other symptoms
D) Brief episodes of freezing, especially when initiating walking
E) A pronounced improvement in motor symptoms after consuming caffeine

A 65-year-old man, recently diagnosed with Parkinson's disease, shows moderate symptoms (such as bradykinesia and rigidity) but no severe cognitive impairment. Which of the following treatments is typically recommended first?

A) Intensive physiotherapy alone
B) A medication that slows disease progression
C) Surgical intervention (deep brain stimulation) for symptom control
D) Dietary supplements and vitamins
E) Medication for symptomatic control

Which of the listed proteins is most important for the function of hemidesmosomes?

A) Integrins
B) Connexins
C) Tubulins
D) Cadherins
E) Claudins

Which statement regarding the zonula adhaerens is most accurate?

A) It is a spot-like cell contact.
B) It closes off intercellular spaces and acts as a barrier.
C) It is formed by cadherins that are connected to actin via catenins inside the cell.
D) It is formed by cadherins that are connected to intermediate filaments via catenins inside the cell.
E) It is formed by integrins that are connected to actin via desmoplakin inside the cell.

Which statement is correct? Glycoproteins and glycolipids of the cell membrane

A) are not determinants of the ABO blood group system.
B) occur only in plants.
C) can only be obtained through diet.
D) are essential components of animal cell membranes.
E) carry polysaccharides only on the cytoplasmic side.

Glycolipids are an important component of the cell membrane. They are formed by attaching sugar residues to ceramides in which organelle?

A) The cell nucleus
B) The lysosome
C) The cell membrane
D) The rough endoplasmic reticulum
E) The Golgi apparatus

You have performed a bedside test with the following result. Which conclusion can you draw from it?

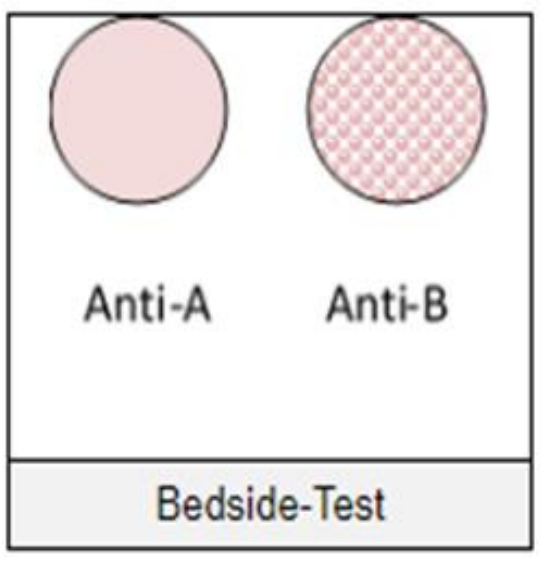

A) The patient has blood type A.
B) The patient has blood type B.
C) The patient has blood type A, Rh+.
D) The patient has blood type A, Rh–.
E) The patient has blood type B, Rh+.

A 55-year-old patient needs a blood transfusion following surgery. To ensure safety and minimize transfusion-related complications, certain checks must be done beforehand. Which step is most crucial for ensuring compatibility and safety?

A) Checking the storage conditions and the expiration date of the blood unit
B) Performing a direct Coombs test to identify autoantibodies
C) Testing donor blood for infectious agents such as HIV, hepatitis B, and hepatitis C
D) Determining the recipient's blood type via a bedside test
E) Measuring the hemoglobin concentration and hematocrit in the blood unit

Which step in the bedside test is critical to ensure patient safety and prevent acute transfusion reactions?

A) Checking the temperature of the blood product before transfusion
B) Matching the patient's identification with the information on the blood product and the transfusion order
C) Verifying medication allergies on the transfusion form
D) Measuring blood pressure immediately before the transfusion
E) Reviewing the current medication regimen to rule out interactions

Which statement about gap junctions is correct?

A) They occur commonly at neuronal synapses.
B) They transmit impulses by releasing neurotransmitters.
C) They connect adjacent cardiac muscle cells.
D) They enable the exchange of proteins between cells.
E) They facilitate paracellular transport.

Which molecule is typically taken up via secondary active transport along with Na+ at the apical membrane of an intestinal epithelial cell?

A) Nitric oxide
B) Glucose
C) Fructose
D) Folic acid
E) Cholesterol

Which type of chromosomal aberration can occur in a fertilized zygote due to errors in chromosome separation during the first or second meiotic division?

A) Triplet expansion
B) Transversion
C) Trisomy
D) Translocation
E) "Trilogy"

Huntington's disease (chorea Huntington) is an autosomal dominant neurodegenerative disorder usually manifesting in mid-adulthood. Which genetic alteration in the huntingtin gene causes this disorder?

A) Deletion
B) Inversion
C) Missense mutation
D) Nonsense mutation
E) Trinucleotide expansion

In the figure, you see a genome arranged via G-banding (karyogram). What is the correct karyotype?

A) 46, XY +13
B) 46, XX +13
C) 47, XY +13
D) 47, XX +13
E) 44, XX +13

Cytogenetics has many medical indications. Which of the following is NOT one of them?

A) Advanced maternal age during pregnancy
B) Determination of the unborn child's sex
C) Dysmorphic features / organ malformations in childhood
D) Leukemia in adulthood
E) Physical or mental developmental delay

What is the empirical probability that a child will have Trisomy 21 if the mother carries a Robertsonian translocation of the type rob(14;21)?

A) 5%
B) 10%
C) 50%
D) 75%
E) 100%

Bacteria can develop resistance to antibiotics. What is the main cause of this?

A) Their low rate of reproduction
B) Transfer of plasmids
C) Formation of bacterial capsules
D) Release of exotoxins
E) Formation of spores

Which of the following features is characteristic of most Gram-negative bacteria?

A) Teichoic acids in the murein layer
B) An outer membrane on top of the murein layer
C) Lipopolysaccharides in the cytoplasmic membrane
D) No murein layer
E) A multilayered murein

Which process do cephalosporins inhibit in bacteria?

A) RNA synthesis
B) DNA synthesis
C) Cell wall synthesis
D) Folic acid synthesis
E) Protein synthesis

A clinical microbiologist explains to a medical student why antibiotic selection should target specific structures in bacterial cells. Which of the following targets is characteristic of an antibiotic that effectively inhibits bacterial protein synthesis and is thus a good choice for treating bacterial infections?

A) Inhibiting folic acid synthesis in bacterial metabolism
B) Blocking bacterial DNA gyrase
C) Binding to the 30S subunit of bacterial ribosomes
D) Inhibiting cell wall biosynthesis by blocking transpeptidase
E) Interfering with bacterial lipid synthesis

A 78-year-old patient with chronic conditions is admitted for a suspected severe community-acquired pneumonia. His CRB-65 score is 4, indicating high mortality risk. According to guidelines, which antibiotic combination is most likely recommended for this patient?

A) IV piperacillin/tazobactam plus a macrolide
B) IV ciprofloxacin plus clindamycin
C) Oral therapy with a macrolide and a beta-lactam
D) Oral therapy with penicillin
E) IV vancomycin plus levofloxacin

A 40-year-old patient with no prior medical history is treated for a suspected bacterial infection. Several principles guide effective and responsible antibiotic therapy. Which principle is most important to minimize antibiotic resistance and maximize effectiveness?

A) Always use the broadest-spectrum antibiotic available.
B) Adjust the dose of the antibiotic based on infection severity.
C) Begin with empirical therapy, then adjust based on culture and sensitivity results.
D) Use combination therapy for every bacterial infection (“Hit hard and early”).
E) Select an antibiotic based on the likely pathogen and local resistance patterns.

A distinction is made between endoparasites and ectoparasites. Which organism is a typical representative of ectoparasites?

A) Plasmodium falciparum
B) Ascaris lumbricoides
C) Trichinella spiralis
D) Ixodes ricinus
E) Echinococcus multilocularis

The malaria pathogen Plasmodium falciparum:

A) is a bacterium
B) causes tertian fever (three-day fever pattern)
C) forms persistent stages in the liver
D) reproduces both sexually and asexually
E) infects humans, gorillas, and chimpanzees

A 35-year-old man is planning a two-month journey through various parts of Southeast Asia, including rural areas in Thailand, Vietnam, and Cambodia. He has no relevant previous illnesses and is vaccinated according to RKI guidelines. He asks for advice on prevention. Which recommendation is most appropriate to minimize infection risk?

A) Take vitamin C and zinc supplements regularly before and during travel
B) Use a DEET-based insect repellent on skin and clothing
C) Avoid meat and animal products entirely
D) Wear long-sleeved clothing at all times, regardless of climate
E) Take antibiotics prophylactically for the entire duration of travel

A 28-year-old woman returns from a four-week backpacking trip in West Africa. Three days later, she develops fever, chills, and severe headaches. A rapid test in your practice is positive for Plasmodium falciparum. Which step is most appropriate immediately after diagnosis?

A) Refer her for inpatient admission and treatment in a specialized center, regardless of symptom severity.
B) Order extensive lab diagnostics, including complete blood count, blood smears for parasites, and kidney function tests, before starting specific treatment.
C) Start empirical IV quinine therapy while arranging referral to a specialized center.
D) Perform a teleconsultation with an infectious disease specialist to discuss outpatient therapy with a suitable antimalarial.
E) Immediately begin oral artesunate therapy on an outpatient basis, provided there are no signs of complicated malaria.

A 26-year-old man presents with intensely itchy skin lesions for about a week. On examination, you find numerous small, raised burrows on the wrists, between the fingers, and

in the axillary region. Based on these clinical signs, you diagnose scabies. Which treatment strategy is most appropriate?

A) A single oral dose of ivermectin
B) Topical application of hydrocortisone cream twice daily for two weeks
C) Whole-body application of 5% permethrin cream for 12 hours
D) Daily application of benzoyl peroxide gel for one week
E) Systemic treatment with a broad-spectrum antibiotic for seven days
