# Supplementary material for: Vertical integration of clinical knowledge in preclinical medical education: assessing the impact on the first-year biology curriculum in a German medical school
Source: BMC Med Educ. 2026 Nov 3;26:1774. doi: 10.1186/s12909-026-10716-7 (PMC13729362; doi:10.1186/s12909-026-10716-7)
Supplement: Supplementary file 3 [file 12909_2026_10716_MOESM3_ESM.pdf]

Please rate the following statements by using:

[completely agree / rather agree / partly/partly / rather disagree / completely disagree]

- The clinical impulses were comprehensible for me in terms of content and the requirements were appropriate.
- I found the clinical impulses to be a valuable addition to the lectures.
- The clinical impulses gave me a good understanding of the importance of the taught biological fundamentals in medicine.
- The clinical contribution was well structured and clearly presented.
- The clinical contribution should be maintained in the future.

Overall evaluation of the clinical contribution

(Please use German school grades 1–5, where 1 = best and 5 = worst.)

Please give us a brief response to the following question.

What did you like about the clinical contribution, and what could be improved?

[open text field]
