# Supplementary material for: Vertical integration of clinical knowledge in preclinical medical education: assessing the impact on the first-year biology curriculum in a German medical school
Source: BMC Med Educ. 2026 Nov 3;26:1774. doi: 10.1186/s12909-026-10716-7 (PMC13729362; doi:10.1186/s12909-026-10716-7)
Supplement: Supplementary file 4 [file 12909_2026_10716_MOESM4_ESM.pdf]

Supplement

Supplementary Table 1: Detailed learning objectives of the conducted curriculum. The curriculum consisted of 6 clinical impulse lectures, each lasting 30 to 45 minutes, integrated into regular biology classes over the course of the first semester. These lectures focused on the clinical application of theoretical biology knowledge.

**Biology**	**Clinical Impulse**
**Living systems and characteristics of life**<br><br>*The students can name the characteristics of living systems and analyse cells in different preparation for these characteristics.*	**Ethics and medicine**<br><br>*The students can deal with ethical questions about the beginning of human life and are familiar with the various arguments in the ethical debate.*
**Proteins - structure and function**<br><br>*The students can describe protein structure and different functions as well as structure-function-relationships.*	**Neurodegenerative diseases**<br><br>*The students can describe the significance of dysfunctional proteins in neurodegenerative diseases, in particular the altered structure and function of alpha-synuclein in Parkinson's disease.*<br><br>*The students can name symptoms and therapeutic interventions in Parkinson's disease.*
**DNA, chromosomes and inheritances**<br><br>*The students can describe the structure and function of the cell nucleus, DNA, chromosomes and the human genome.*<br><br>*The students can describe the process of meiosis and its stages as well as chromosomal maldistributions due to non-disjunction or structural changes caused by various noxious agents.*	**Cytogenetics**<br><br>*The students can explain numerical as well as structural changes in the genome, their causes and apply their knowledge to clinical cases (karyogram).*<br><br>*The students can describe the key stages of the process, from the indications for cytological diagnostics, anamnesis and blood collection, through laboratory and resulting report.*
**Blood cells and glycocalyx**<br><br>*The students can explain the structure and function of erythrocyte as well as the glycocalyx and its relevance for the ABO-blood system.*	**Transfusion medicine**<br><br>*The students can perform a bedside test under supervision and explain its medical relevance in the context of blood transfusions.*<br><br>*The students can describe when blood transfusions are used, name important legal bases as well as responsibilities and can explain actions to prevent incorrect transfusions.*
**Bacteria and antibiotics**<br><br>*The students can explain the structure of the prokaryotic cell and the classification criteria of bacteria.*<br><br>*The students can explain different cell targets of antibiotics and can name different classes as well as substances.*	**Antibiotics**<br><br>*The students can describe different strategies for the use of antibiotics in clinical practice and apply these to a specific case (pneumonia)*
**Parasitology**<br><br>*The students can explain the term parasitism as well as the categorization and morphology of human pathogenic parasites.*<br><br>*The students can describe the principles of parasite reproduction, including different stages of development, vectors and hosts.*	**Clinical parasitology**<br><br>*The students can transfer their theoretical knowledge to clinical praxis and describe symptoms and therapy für malaria infection, scabies and Lyme borreliosis.*

Supplementary Table 2: Comparison of basic participant characteristics between subgroups examined in subgroup analyses. Significant values are highlighted in bold.

**Item**	**Group 1 (n = 11)**	**Group 2 (n = 94)**	**P-value**
Age, mean (SD) [years]	21 (3.4)	22.4 (3.3)	0.189
Sex, male number, N (%)	1 (9)	29 (30.9)	0.172
HS-GPA, mean (SD)	1.39 (0.44)	1.61 (0.55)	0.218
Previous medical education, N (%)	4 (36.4)	50 (53.2)	0.35
Biology at school, N (%)			0.49
Basic level	7 (64)	40 (43)	
Advanced level	3 (27)	45 (48)	
Neither basic nor advanced	0 (0)	3 (3)	
Not during A-levels	1 (9)	6 (6)	
Other	0 (0)	0 (0)	

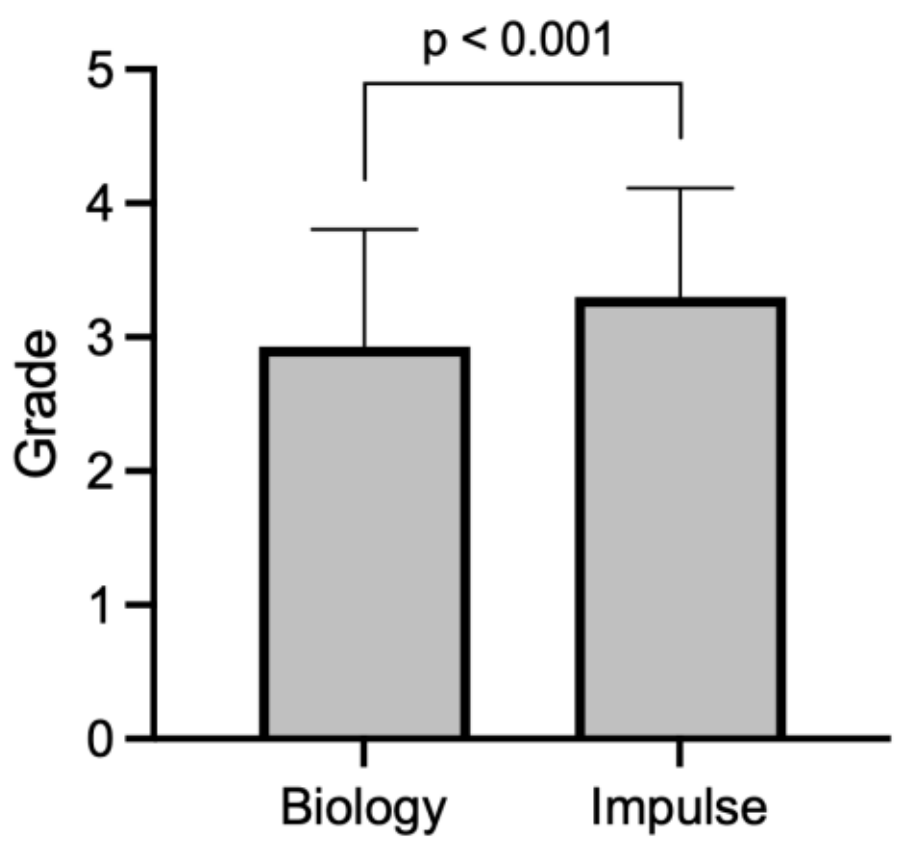

Supplementary Figure 1: Comparison of the baseline self-assessment of biology and impulse knowledge. Grades for the students' self-assessment of both biology and clinical impulse knowledge were compared for the baseline evaluation. Statistical analysis was conducted using an unpaired student's t-test.

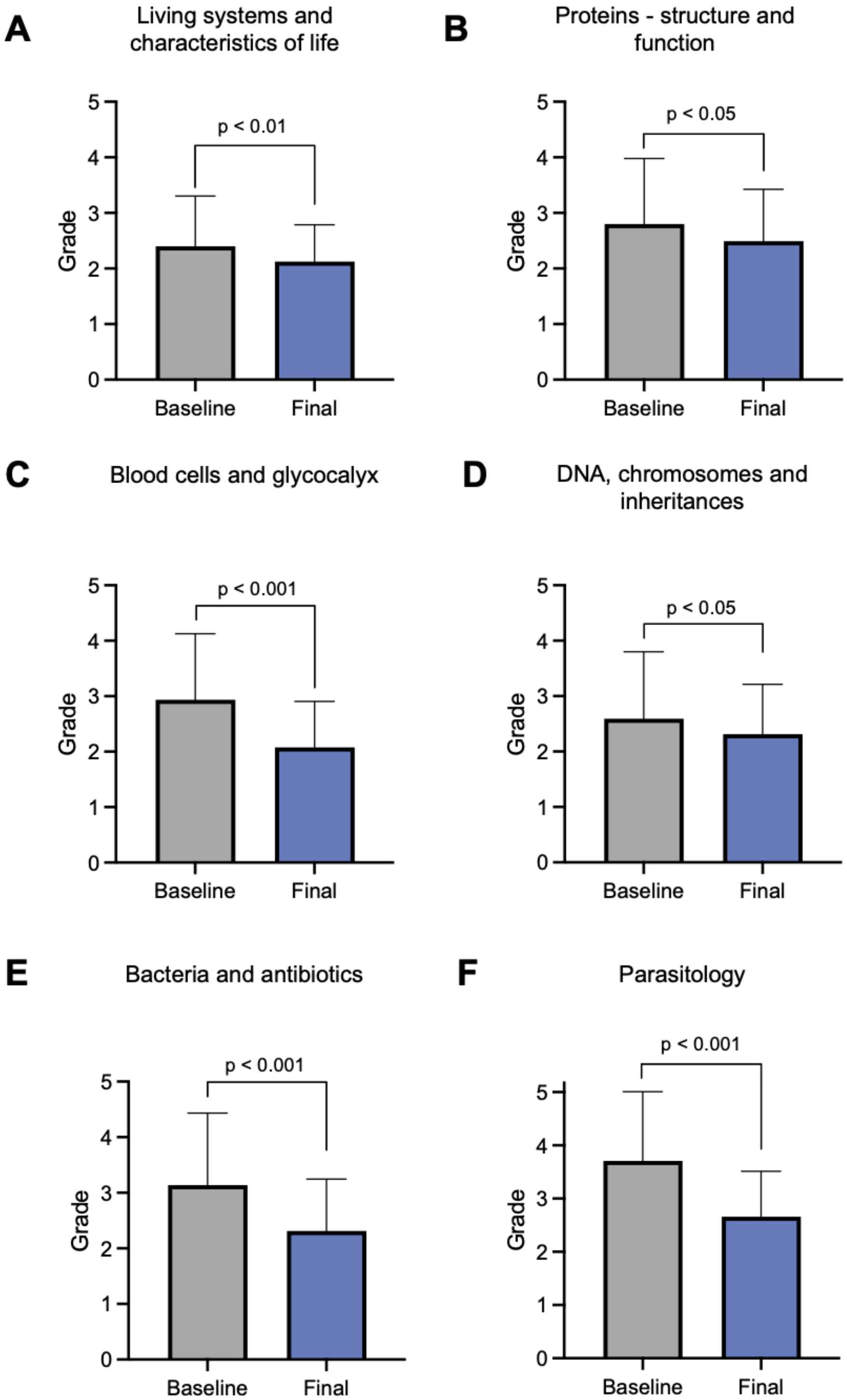

Supplementary Figure 2: Impact of the curriculum on the self-assessed biological knowledge for each topic. The grade for the self-assessment of biological knowledge is depicted for each topic of the curriculum for both the baseline and final evaluation. Statistical analysis was conducted using an unpaired student's t-test.

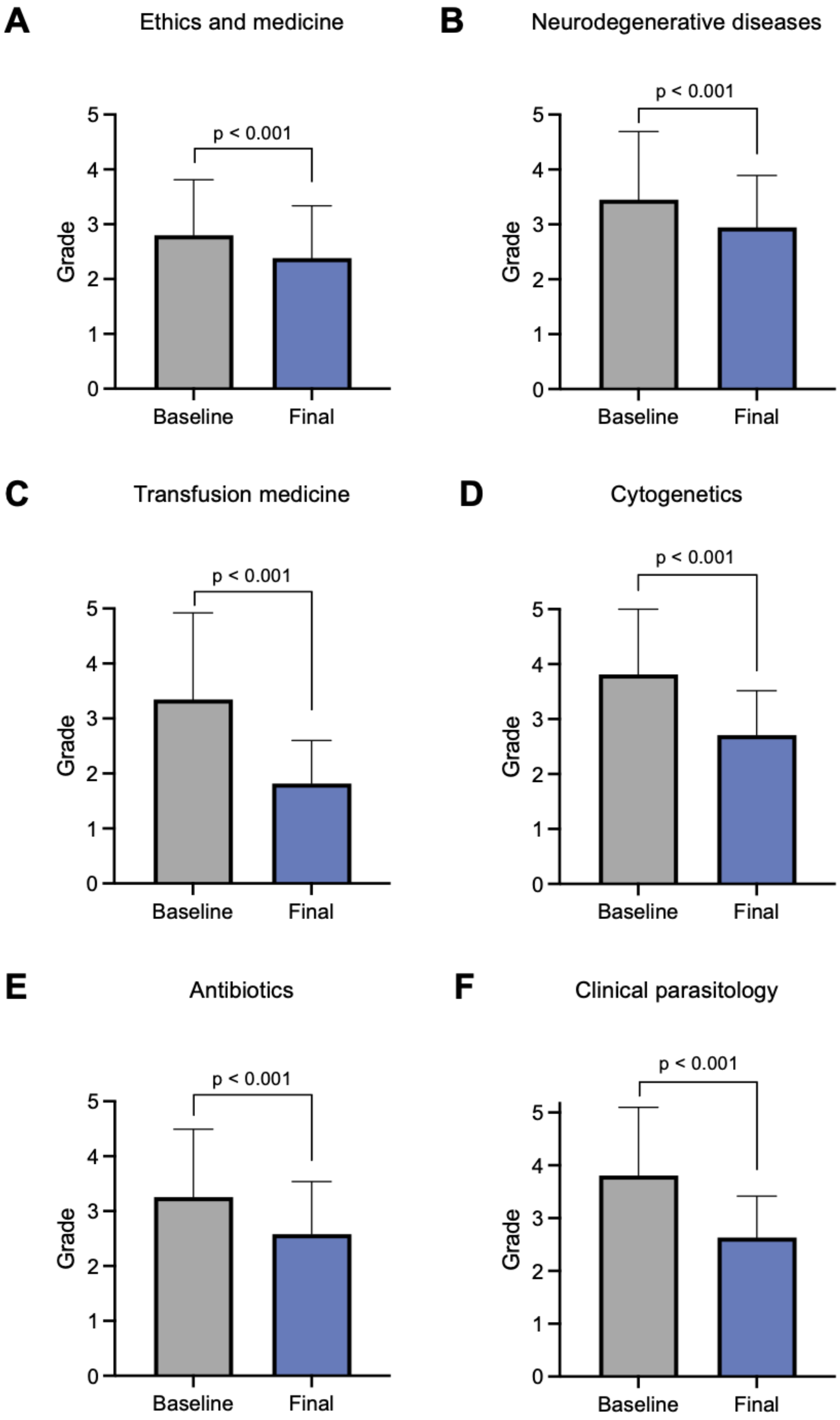

Supplementary Figure 3: Impact of the curriculum on the self-assessed knowledge for each impulse. The grade for the self-assessment of the clinical impulse knowledge is depicted for each impulse topic of the curriculum for both the baseline and final evaluation. Statistical analysis was conducted using an unpaired student's t-test.
